# Marketing Two Immunization Services at a Regional Supermarket Chain Pharmacy

**DOI:** 10.3390/pharmacy11030103

**Published:** 2023-06-15

**Authors:** Megan A. Hedden, Yifei Liu, Peggy G. Kuehl, Sarah M. Oprinovich

**Affiliations:** 1 Barry’s Drug Center, Manhattan, KS 66502, USA; 2 Division of Pharmacy Practice and Administration, University of Missouri—Kansas City School of Pharmacy, Kansas City, MO 64108, USA

**Keywords:** personal selling, automated telephone call, immunization, pharmacist, pneumococcal disease, herpes zoster

## Abstract

*Background*: Personal selling of immunization services includes starting a dialogue with patients, utilizing effective questioning and listening skills to identify their vaccination needs, and recommending appropriate vaccines accordingly. The study objectives were (1) to integrate personal selling into the dispensing workflow to promote pneumococcal polysaccharide vaccine (PPSV23), and (2) to evaluate the impact of personal selling and automated telephone calls to promote herpes zoster vaccine (ZVL). *Methods*: For the first study objective, a pilot project was conducted at one out of 19 affiliated supermarket pharmacies. Dispensing records were used to target patients with diabetes mellitus for PPSV23, and personal selling was implemented over a 3-month period. For the second study objective, a full study was conducted among the nineteen pharmacies, with five in the study group and 14 in the control group. Personal selling was implemented over a 9-month period, and automated telephone calls were placed and tracked over a 6-week period. Mann–Whitney U tests were used to compare vaccine delivery rates between the study and control groups. *Results*: In the pilot project, 47 patients needed PPSV23, but none received it from the pharmacy. In the full study, 900 ZVL vaccines were given, with 459 given for 15.5% of the eligible patients in the study group. During the time when 2087 automated telephone calls were placed and tracked, 85 vaccines were given across all pharmacies, with 48 given for 1.6% of the eligible patients in the study group. During both the 9-month and 6-week periods, the mean ranks of vaccine delivery rates in the study group were higher than the control group (*p* < 0.05). *Conclusions*: The pilot project incorporated personal selling into the dispensing workflow and, although no vaccines were given, provided valuable lessons. The full study demonstrated that personal selling alone and personal selling combined with automated telephone calls were associated with higher vaccine delivery rates.

## 1. Introduction

In the U.S., community pharmacists are an integral part of the healthcare team and play a critical role in patient care, particularly in the provision of immunization services. Community pharmacists are well-positioned to provide immunization services by often offering easier access to vaccines than traditional immunization settings, longer hours of availability, and locations close to patients’ homes [1,2]. In addition, community pharmacists have demonstrated a positive impact on vaccine uptake [3,4,5]. Today, pharmacists are authorized to provide immunizations in all 50 U.S. states [6], and community pharmacies routinely provide immunization services. To be efficient immunizers and provide financially sustainable services, community pharmacists need to engage in marketing efforts to raise awareness among patients, target patients at risk for a vaccine-preventable illness, and promote immunization services.

Personal selling is a promotion strategy that pharmacists can use to market pharmacy services [7]. In terms of promoting immunization services, personal selling requires pharmacists to initiate a conversation with the patient about vaccinations, use effective questioning and listening skills to identify patients’ specific health needs, and present relevant vaccines that meet those needs [8,9]. A pharmacist can initiate a conversation by greeting patients warmly and asking open-ended questions about their health. These questions may include inquiring about symptoms patients are experiencing, their medical history, and medications they are taking [7]. By asking open-ended questions, the pharmacist can encourage detailed answers to identify patients’ specific health needs. The pharmacist should be able to communicate the benefits of the recommended immunization services in a clear and concise manner and ensure that patients understand the benefits. In addition, personal selling provides the pharmacist with an opportunity to address potential barriers to accessing immunization services [8]. For example, patients may be reluctant to receive immunizations due to concerns about cost or unclear recommendations from their healthcare providers. Pharmacists can help determine the patient’s out-of-pocket costs or educate the patient on current vaccine recommendations and benefits. The pharmacist’s role in addressing these barriers is critical for facilitating patient engagement and improving overall health outcomes.

Automated telephone calls have emerged as another tool to engage patients in various health behaviors [10]. Some automated telephone calls use interactive voice-response (IVR) systems [11]. This technology can provide reminders for upcoming appointments, directions to the healthcare facility, and scheduling options. Patients can respond by pressing buttons on their telephone keypads. One study reported that IVR telephone calls significantly improved the likelihood among patients to receive colorectal cancer screenings [12]. Another study used IVR telephone calls to administer the Short Inflammatory Bowel Disease Questionnaire and verified that the results were similar to written self-administration [13]. Some automated telephone calls use automated voice messages, a one-way communication channel that delivers standardized messages without the ability to respond [14]. Community pharmacists can consider using automated telephone calls as part of their marketing strategies to promote immunization services.

Although personal selling and automated telephone calls are commonly used in marketing, evidence is needed on their role in promoting immunization services in the community pharmacy setting. The objectives of this study were (1) to integrate personal selling into the dispensing workflow to promote an immunization service for pneumococcal polysaccharide vaccine (PPSV23), and (2) to evaluate the impact of personal selling and automated telephone calls to promote an immunization service for Zostavax^®^ (ZVL). ZVL is a vaccine that is currently discontinued in the U.S., but was recommended by the Centers for Disease Control and Prevention (CDC) for the prevention of herpes zoster in adults 60 years of age or greater at the time of study [15].

## 2. Methods

### 2.1. Practice Setting

At the time of study, Balls Food Stores was a regional supermarket chain consisting of 19 pharmacies in the Kansas City metropolitan area under Price Chopper and Hen House banners. We conducted a pilot project at one pharmacy for the first study objective, and a full study at all 19 pharmacies for the second study objective. According to the CDC, PPSV23 is recommended for two groups of individuals—all individuals 65 years of age or older, and individuals 2 years of age or older with certain medical conditions who have higher risk of developing pneumococcal disease [16]. In addition, the Advisory Committee on Immunization practices of the CDC recommends Shingrix^®^ for adults 50 years and older to prevent herpes zoster [17]. However, the full study occurred when ZVL was available and Shingrix^®^ was not. Before Shingrix^®^ was introduced, ZVL was recommended for adults 60 years of age and older [15].

The immunization program at Balls Food Stores began with the use of a contract group of nurses to provide immunization services. Later, Balls Foods Stores obtained a contract with Medicare Part B to provide vaccines to covered patients. In the pilot project, Balls Food Stores was transitioning from hosting nurse-led immunization clinics to a pharmacist-run immunization program, and one pharmacy had begun offering PPSV23 on a walk-in basis. The full study was conducted after Balls Food Stores established the pharmacist-run immunization program.

### 2.2. Pilot Project

The pilot project was conducted at a single pharmacy. Dispensing records were used to identify patients with diabetes mellitus, who therefore had an indication for PPSV23. Diabetes mellitus was chosen because the medications used were specific to the disease state with relatively few exceptions. All oral hypoglycemic agents, all types of insulin, glucagon-like peptide agonists, and pramlintide were used to identify patients. The pharmacy dispensing software was used to generate reports of patients who had a prescription for these medications in the previous 90 days. All patients who were at least 18 years of age, and who had at least one of these medications, were included. Personal selling was implemented over a 3-month period, and scripts were developed for both personal selling and for obtaining the patient’s verbal consent. Pharmacists and technicians were alerted to eligible patients by flagging the prescriptions. When a patient arrived to pick up a flagged prescription medication, the staff used the script and asked the following questions:

*“(1) Have you been diagnosed with diabetes?; (2) Have you received a pneumonia shot?; (3) Where did you get your shot?; (4) What year did you get your shot?”*

If a patient had a diagnosis of diabetes but had not received PPSV23 or had an indication for revaccination, then the pharmacist encouraged the patient to get the vaccine either immediately or by appointment. The number of patients vaccinated, the number of targeted patients with an indication for PPSV23, and responses from patients who needed PPSV23 were reported. Approval for the pilot project was granted by the University of Missouri—Kansas City Social and Behavioral Sciences Institutional Review Board (IRB).

### 2.3. Full Study

The full study involved all 19 pharmacies, with five serving as the study group and the remaining 14 serving as the control group. The five pharmacies in the study group promoted the immunization service for ZVL by implementing personal selling as well as automated telephone calls. These five pharmacies were selected as the study group because they had adequate freezer space to store the vaccine, making them suitable for providing the vaccine to patients on a walk-in basis.

Personal selling was implemented over a 9-month period. One month before the promotion of the immunization service, personal selling training was provided by a consultant to three in-house pharmacy staff trainers. The training consisted of a one-hour didactic lecture with active-learning components and role playing. A script to address anticipated patient concerns regarding receipt of the vaccine was provided (Table 1).

These in-house trainers, in turn, provided on-site training to the pharmacy staff at the five pharmacies. In addition, the trainers provided weekly coaching to help each pharmacy achieve its personal selling goals. Automated telephone calls were added for two weeks during the eighth month of the personal-selling implementation. The calls were tracked for four weeks after they were made to assess the effects, because most patients visit the pharmacy at least once per month. If they were waiting for a convenient time to come in (e.g., when they picked up their prescription medications), one month would allow for enough time to capture patients who wanted the vaccine. So, two different study periods were used to distinguish between the combination of personal selling and automated telephone calls (6 weeks) from personal selling alone (9 months).

In the study group, each pharmacy identified patients who were at least 60 years old by tagging their prescriptions with a bag tag. This step was performed by a pharmacy technician. Again, the age criterion was based on the CDC’s recommendation at the time of study. During pick-up, the bag tag prompted pharmacy staff to engage in personal selling to determine the patient’s need for ZVL. If the patient was over 60 and had not received the vaccine, then a pharmacist was called over to discuss eligibility for the vaccine with the patient. Each pharmacy was given a script for addressing patient barriers to receiving the vaccine and for guiding the personal selling process. The number of bag tags completed was also used as a measure of the pharmacy staff’s use of personal selling, with each pharmacy having a goal of completing 12 bag tags and administering five vaccines per week.

Automated telephone calls were used to further raise patient awareness of their eligibility for ZVL. To do this, the dispensing software was used to target patients at each pharmacy who met the following criteria: (1) the patient had to be at least 60 years old; (2) the patient must have filled a prescription at one of the five pharmacies within the previous 90 days; (3) the patient must have not already received ZVL at the pharmacy. Automated telephone calls were placed to 25% of the eligible patients at each pharmacy on four separate dates (thus reaching 100% of eligible patients) which were spread out over two weeks. This was to prevent such an increase in vaccine requests that might overwhelm the pharmacy staff and create a possible vaccine shortage. Personal selling continued during the six weeks of placing and tracking automated telephone calls. The message delivered by the automated phone call was as follows.

*“This is your (Price Chopper/Hen House) Pharmacy located at (XXXXX) calling to let you know we have the Zostavax^®^ vaccine available and that someone in your household may benefit from receiving Zostavax^®^, the vaccine that prevents shingles. To make an appointment or find out more information about receiving the shingles vaccine, please call us or stop by the pharmacy. If you have any questions, please call us at (XXX-XXX-XXXX). Thank you for choosing your (Price Chopper/Hen House) Pharmacy. Good bye”.*

Data were collected for each pharmacy retrospectively to determine the number of eligible patients before the study period and the number of ZVL vaccines administered during the study period. Vaccine delivery rate was calculated as the number of vaccines administered during the corresponding study period divided by the number of eligible patients at each pharmacy. In addition, Kolmogorov–Smirnov and Shapiro–Wilk tests were used to test the normality of vaccine delivery rates over the 9-month period of implementing personal selling and the 6-week period of placing and tracking automated telephone calls. Based on the results, appropriate statistical tests were selected to compare vaccine delivery rates between the study and control groups. Of note, an economic analysis of the immunization service for ZVL was reported elsewhere [9]. In this study, we focused on evaluating the outcomes of the service in terms of marketing. Approval was granted for the full study by the University of Missouri—Kansas City Social and Behavioral Sciences IRB.

## 3. Results

In the pilot project, the pharmacy dispensed an average of 743 prescriptions per week, and 138 patients received medication to treat diabetes. Of these patients, one patient was less than 18 years old, one was deceased, three were no longer customers of the pharmacy, 16 denied having a diagnosis of diabetes, 12 could not be contacted, and one did not consent to participate. Therefore, the final analysis included 104 patients. Forty-seven (45.2%) patients needed PPSV23, but none received it at the pharmacy. One patient received PPSV23 from a physician. Of the 47 patients, 18 offered reasons for not receiving the vaccine, ten patients refused the vaccine, one believed not to be susceptible to pneumonia, and another reported a desire to wait for Medicare to cover the vaccine.

In the full study, all 19 pharmacies filled a total of approximately 13,110 prescriptions per week, with an average of 690 (range: 415–1322) prescriptions per pharmacy per week. There were 9920 patients eligible for ZVL, with an average of 522 (range: 185–1026) eligible patients per pharmacy. During the nine-month period of implementing personal selling, 900 ZVL vaccines were given across all 19 pharmacies with 459 vaccines given at the 5 pharmacies performing personal selling (Table 2). Of the eligible patients, 9% were vaccinated across all pharmacies during the implementation of personal selling, whereas 15.5% of the eligible patients were vaccinated in the study group.

A total of 2087 automated telephone calls were placed. Of these, 1915 calls “passed”, meaning that they either connected with an actual person or a message was left in a voicemail inbox. The remaining 172 calls “did not pass”, meaning that either the phone line was busy, there was an error with the telephone number (e.g., it had been disconnected, was no longer in service, etc.), a fax tone was reached, or the call was not answered by a person or voicemail (Table 3). During the time when the automated telephone calls were placed, 85 vaccines were given across all 19 pharmacies, with 48 vaccines given at the five pharmacies in the study group.

Vaccine delivery rates during both periods deviated from a normal distribution (*p* < 0.05). Therefore, Mann–Whitney U tests were used to compare vaccine delivery rates between the study and control groups (Table 4). The mean ranks in the study group were higher than the control group in either test (*p* < 0.05).

## 4. Discussion

Although the pilot project incorporated personal selling into the dispensing workflow, no patients received PPSV23 from the pharmacy. This highlights the importance of addressing patient barriers to immunization. One study examined the barriers faced by patients and healthcare providers to receiving vaccines, particularly tetanus, influenza, and pneumococcal vaccines [18]. The two most common reasons patients did not receive PPSV23 were the belief that a healthy person did not need the vaccine and because the patients’ healthcare providers had not recommended it. Interestingly, healthcare providers cited different reasons than those reported by patients, such as inadequate insurance coverage for PPSV23. The study suggests that patient education and reminders can overcome patient barriers. Other studies also support the notion that patient education influences a patient’s decision to receive a vaccine [19,20]. Perhaps the amount of time spent educating patients in the pilot project was not enough for patients to seek PPSV23. In addition, although pharmacy staff were instructed on how to deliver the point-of-care intervention, no script was provided to address potential patient barriers to receipt of the vaccine. Similar to a previous study [21], we found personal selling to be difficult or uncomfortable for pharmacists. It was unclear how persuasive pharmacy staff were or how they customized personal selling to each patient. More effective use of personal selling may have resulted in greater interest in receiving PPSV23. Nevertheless, the pilot project provides valuable lessons on how to improve the implementation of personal selling. For example, the full study included personal-selling training, use of a script, weekly coaching, and weekly personal-selling goals.

The full study shows that personal selling alone and personal selling combined with automated telephone calls increased ZVL delivery rates in the study group. Vaccine delivery rates were highest at Hen House 32 (Table 2 and Table 3). This may be because Hen House 32 is located in a suburban area with a younger population; thus, the number of eligible patients (n = 185) was smaller than the other four pharmacies in the study group. However, the focus of this study was on two groups of pharmacies rather than individual locations. Future research could examine other characteristics of individual pharmacies with higher vaccine delivery rates. The mean ranks of vaccination delivery rates of the study group in Mann–Whitney U tests were higher (Table 4), which indicates the effectiveness of the two marketing techniques. Community pharmacies are increasingly involved in the provision of immunization services [22]. Such involvement not only benefits patients, but also provides an alternative source of revenue for community pharmacies. In the current climate of declining third-party reimbursement and rising prescription costs, it can be challenging to sustain pharmacy operations. However, personal selling and automated telephone calls can be used to promote pharmacy services, including immunization services, enabling community pharmacies to remain profitable and continue to provide essential healthcare services to patients. Interestingly, a randomized controlled trial found no effect of an automated telephonic prompt on vaccination rates, with barriers including inadequate pharmacy follow-up and low patient priority for vaccination [23]. In the full study, we did not use automated telephone calls alone but in combination with personal selling. Personal selling can complement automated telephone calls to address these barriers.

In both the pilot project and the full study, dispensing records were used to identify patients at risk for vaccine-preventable diseases. This method of searching computerized dispensing records was efficient. Manual chart reviews and pharmacy database searches are two methods to identify at-risk patients [18,24]. In one study, a pharmacy database was searched for patients taking medications that would indicate a need for a vaccine, and then chart reviews were conducted to screen for vaccine indications and contraindications [24]. In both the pilot project and the full study, pharmacy staff did not have access to medical records. Pharmacists relied on patients to provide accurate information about a vaccine’s indications and contraindications. However, because the patients themselves were screened during personal selling, as opposed to reviewing their medical records, the process can be integrated into the dispensing workflow without requiring additional staff time.

Both the pilot project and the full study were conducted at Balls Food Stores, a regional supermarket chain in Missouri and Kansas. This limits the generalizability of the results. In the pilot project, recall bias from patients could occur when asking about the diagnosis of diabetes. In addition, there were four limitations in the full study. First, seven of the 19 pharmacies did not have a freezer for storing the vaccine during the study period. Thus, the seven pharmacies that did not have freezers were unable to provide the vaccine to patients on a walk-in basis. To overcome this challenge, these pharmacies collected the names of patients interested in receiving ZVL and scheduled appointments to provide it. On the scheduled day, a pharmacist would come to the pharmacy with the vaccine on dry ice and administer multiple injections in one day. Those pharmacies gave fewer vaccinations compared to those that had freezers. This issue highlights the importance of proper storage to ensure vaccines are readily available to patients, as well as efficient scheduling and timely delivery of vaccines to meet patient needs. Second, there was pharmacist turnover in one of the five pharmacies where personal selling was implemented. This pharmacy had the lowest number of ZVL administered during the implementation period of personal selling. The new pharmacist was never trained in personal selling due to scheduling conflicts. Third, despite the use of automated telephone calls to reach eligible patients, not all individuals were successfully contacted. Of the 2087 calls made, 172 were unsuccessful in reaching the intended recipients. No further attempts were made to contact these individuals, which could have resulted in missed immunization opportunities. Lastly, when determining eligible patients for automated telephone calls, only patients who received ZVL at that particular pharmacy were excluded due to limitations of the automated system. For example, an individual patient may have been on the call list for two different pharmacies and was excluded at one pharmacy, because the patient received the vaccine there before the start of the study, but still remained on the call list at the second pharmacy and was called. Because ensuring effective communication with patients facilitates their access to health services [25], it is therefore necessary to establish communication channels in addition to telephone calls.

## 5. Conclusions

The pilot project incorporated personal selling of an immunization service into the dispensing workflow and provided valuable lessons in implementing personal selling. The full study demonstrates that personal selling alone and personal selling combined with automated telephone calls were two marketing strategies associated with higher vaccine delivery rates. Personal selling and automated telephone calls can be applied to promote pharmacy services.

## Figures and Tables

**Table 1:** Script of the full study to address patient concerns.

Patient Concerns	Pharmacist’s Responses
“I don’t know if my doctor wants me to get this vaccine”.	“We only provide this vaccine to those patients who meet the requirements set forth by the Centers for Disease Control. We have a protocol with a local physician so we can administer it to you. Would you like us to see if you should receive this vaccine?” *
“I don’t know how much it will cost”.	“We can check with your insurance and verify your copay. Would you like us to do that?” (A pharmacy technician runs a claim for pharmacist to which pharmacist can refer.)
“I don’t have time to get this vaccine right now”.	“We can set up an appointment so you can come in when you have more time”.
“I’m not sure I want to get it today”.	Provide an informational brochure. “Take this home and discuss it with your family. Feel free to call us if you have any questions. We will ask you about it again when we see you again”.

* If patient is adamant about whether or not their primary care physician recommends it, offer to check with primary care physician and call patient with response.

**Table 2:** Results of implementing personal selling in the study group during the 9-month period.

Pharmacy Number	Number of Patients Vaccinated	Number of Eligible Patients	Vaccine Delivery Rate (%)
Hen House 27	85	712	11.9%
Hen House 29	105	728	14.4%
Hen House 32	158	185	85.4%
Price Chopper 36	67	656	10.2%
Price Chopper 42	44	681	6.4%
Total	459	2962	15.5%

**Table 3:** Results of placing automated telephone calls in the study group during the 6-week period.

Pharmacy Number	Pass		Did Not Pass	Total Calls	Number of Patients Vaccinated	Number of Eligible Patients	Vaccine Delivery Rate (%)
	Person	Machine					
Hen House 27	308	129	38	475	6	712	0.8%
Hen House 29	312	170	42	524	11	728	1.5%
Hen House 32	100	64	20	184	8	185	4.3%
Price Chopper 36	259	129	33	421	13	656	2.0%
Price Chopper 42	286	158	39	483	10	681	1.5%
Total	1265	650	172	2087	48	2962	1.6%

**Table 4:** Comparison of vaccination delivery rates between the study and control groups.

Mean Rank of Vaccine Delivery Rate			
Group	During the 9-Month Period of Implementing Personal Selling	During the 6-Week Period of Placing and Tracking Automated Telephone Calls	*p*-Value ^a^
Study group (n = 5)	14.8	14.6	<0.05
Control group (n = 14)	8.3	8.4	<0.05

^a^ Mann–Whitney U tests.

## Data Availability

Data sharing is not applicable to this article.
